# A Framework for the Next Generation of Risk Science

**DOI:** 10.1289/ehp.1307260

**Published:** 2014-04-11

**Authors:** Daniel Krewski, Margit Westphal, Melvin E. Andersen, Gregory M. Paoli, Weihsueh A. Chiu, Mustafa Al-Zoughool, Maxine C. Croteau, Lyle D. Burgoon, Ila Cote

**Affiliations:** 1 McLaughlin Centre for Population Health Risk Assessment, University of Ottawa, Ottawa, Ontario, Canada; 2 Risk Sciences International, Ottawa, Ontario, Canada; 3 Institute for Chemical Safety Sciences, The Hamner Institutes for Health Sciences, Research Triangle Park, North Carolina, USA; 4 National Center for Environmental Assessment, U.S. Environmental Protection Agency, Washington, DC, USA

## Abstract

Objectives: In 2011, the U.S. Environmental Protection Agency initiated the NexGen project to develop a new paradigm for the next generation of risk science.

Methods: The NexGen framework was built on three cornerstones: the availability of new data on toxicity pathways made possible by fundamental advances in basic biology and toxicological science, the incorporation of a population health perspective that recognizes that most adverse health outcomes involve multiple determinants, and a renewed focus on new risk assessment methodologies designed to better inform risk management decision making.

Results: The NexGen framework has three phases. Phase I (objectives) focuses on problem formulation and scoping, taking into account the risk context and the range of available risk management decision-making options. Phase II (risk assessment) seeks to identify critical toxicity pathway perturbations using new toxicity testing tools and technologies, and to better characterize risks and uncertainties using advanced risk assessment methodologies. Phase III (risk management) involves the development of evidence-based population health risk management strategies of a regulatory, economic, advisory, community-based, or technological nature, using sound principles of risk management decision making.

Conclusions: Analysis of a series of case study prototypes indicated that many aspects of the NexGen framework are already beginning to be adopted in practice.

Citation: Krewski D, Westphal M, Andersen ME, Paoli GM, Chiu WA, Al-Zoughool M, Croteau MC, Burgoon LD, Cote I. 2014. A framework for the next generation of risk science. Environ Health Perspect 122:796–805; http://dx.doi.org/10.1289/ehp.1307260

## Introduction

Protecting and enhancing population health is of concern to everyone. By almost any standard, people worldwide are living longer and healthier than ever before. Much of this success can be attributed to effective public health practices that have evolved over the last two centuries, and to the adoption of healthier lifestyles. More recently, powerful techniques in risk science—a term used here to encompass both the scientific enterprise of risk assessment and, in analogy to management science, risk management actions taken to reduce risk—have been used to address important population health risk issues.

Although risk science is now a well-established transdisciplinary field of investigation, the manner in which population health risks are assessed and managed continues to evolve. Historically, risk science has focused on the large number of chemical substances present in the human environment, defined in the broad sense as air, food, water, soil, and the built environment. Today, however, methods in risk science are widely applied in addressing other risk issues of importance to society, including those of a biological or social nature.

Motivated by a number of advances in risk science discussed below, the U.S. Environmental Protection Agency’s (EPA) NexGen project was initiated to design the next generation of risk science, with the specific goal of making risk assessments faster, less expensive, and more scientifically robust (Cote et al. 2012). Chemical risk assessment in particular faces a number of challenges, including the large backlog of untested chemicals, the current movement away from *in vivo* toxicity testing and the prospect of high volumes of *in vitro* toxicity test data, and the desire to consider nonchemical stressors in the risk assessment process. These challenges overlap with the need to increase the quality and utility of risk assessment information in order to provide a solid evidentiary base that will permit choosing among regulatory and other risk management options available to decision makers.

The NexGen framework that we present here is designed to articulate guiding principles that respond to both the challenges and opportunities currently facing risk science. The framework is structured to support decision making, with up-front consideration of a broad array of risk management options. The framework places a strong emphasis on problem formulation to ensure that the risk assessment phase is designed to support a rational choice of decision-making options available to the decision maker within a particular risk context. The framework includes active consideration of determinants of population health and their interactions, ideally before the design of specific testing strategies. At the core of the framework are new risk assessment methodologies to incorporate *in vitro* and *in silico* evidence to enable an improved understanding of toxicity pathways—defined by the National Research Council (NRC 2007) as “normal cellular response pathways that are expected to result in adverse health effects when sufficiently perturbed”—within the classical risk assessment paradigm.

The NexGen framework is based on three cornerstones: *a*) the toxicity pathway–based approach to risk assessment elaborated by the NRC (2007) in its vision for the future of toxicity testing; *b*) a population health approach to risk assessment, taking into account multiple determinants of health and their interactions (Chiu et al. 2013; Krewski et al. 2007); and *c*) the emergence of new risk assessment methodologies, such as those described by the NRC in the “Silver Book,” *Science and Decisions: Advancing Risk Assessment* (NRC 2009). Here we describe the complementary nature of the three perspectives characterizing the NexGen framework for risk science—a pathway-based toxicity testing paradigm, a population health approach, and advanced risk assessment.

## Toxicity Testing in the 21st Century

The first cornerstone, and the primary driver for the NexGen framework, stems from the NRC report, *Toxicity Testing in the 21st Century: A Vision and a Strategy* (NRC 2007), now commonly referred to as *TT21C*. The report recommends an overhaul of the scientific tools and technologies that form the basis for toxicity testing and risk assessment. The shift in focus away from apical end points identified in *in vivo* toxicity tests conducted in mammalian systems toward the use of *in vitro* assays to identify perturbations of toxicity pathways at the molecular and cellular level leading to adverse outcomes will take up to a decade or more to complete, validate, and implement. The overall goal of the vision is to move toxicity testing toward a more evidence-based framework where decisions made regarding risks are based on scientific facts derived from a solid foundation of understanding the signaling pathways involved in both homeostasis and disease, the chemical and molecular events involved in those pathways, and the mechanism of action of the chemical or its metabolites. Understanding human disease pathways at the molecular level—a challenge recently considered by the NRC in *A Framework for Developing a New Taxonomy of Disease* (NRC 2011b)—will also help in understanding toxicity pathways.

*TT21C* will involve the application of a wide array of scientific tools and technologies, including those summarized in Supplemental Material, Table S1 (Andersen et al. 2010; Krewski et al. 2011). These new testing approaches will involve complex end points such as signature profiles and biomarkers of *in vitro* pathway perturbations, more relevant exposure assessments, population-based studies with molecular and genetic components, and the use of predictive toxicity algorithms based on computational systems modeling. Support for this pathway-based approach to toxicity testing has been expressed by Collins et al. (2008) and Hamburg (2011). A consortium of U.S. federal agencies (the *Tox21* program) including the U.S. EPA Office of Research and Development, the U.S. National Toxicology Program, the National Center for Advancing Translational Sciences, and the U.S. Food and Drug Administration (FDA) has been established to advance the *TT21C* vision in a coordinated fashion. The U.S. EPA has elaborated a strategic plan for evaluating the toxicity of chemicals, which outlines a pragmatic stepwise approach to the transition from *in vivo* to *in vitro* testing strategies (U.S. EPA 2009). The plan focuses on using chemically induced mechanisms of action to prioritize chemicals and developing toxicological models to predict human response to chemicals. Chiu et al. (2013) provided additional detail on timelines and the steps needed to transition from risk assessments based on *in vivo* data to primary reliance on high throughput/high content pathway and biomarker data.

*TT21C* has caught the imagination of researchers and regulators internationally. European initiatives such as AXLR8 (AXLR8 2012) and the 7th amendment to the European Union cosmetics directive of 2003 (European Commission 2013) emphasize the use of *in vitro* methodologies in risk assessment (Hartung et al. 2011). Canada has also identified *in vitro* testing as a promising tool in pesticide regulation (Council of Canadian Academies 2012).

## A Population Health Perspective

The second cornerstone of the NexGen framework considers risk from a broader population health perspective, simultaneously examining multiple determinants of health that interact in complex ways to determine population health status. The World Health Organization (WHO 1948) has adopted a broad definition of health as being “a state of complete physical, mental and social well-being and not merely the absence of disease or infirmity.” The health of individuals and populations is not determined by any one factor, but by a complex number of factors that interact with each other. A state of health is a matter of both circumstances and environment. To a greater or lesser extent, factors such as where we live, our environment, genetics, income and education, and behavior all have considerable influence on health (Evans and Stoddart 1990).

The field of population health has been advanced through the work of the Canadian Institutes of Advanced Research (CIAR), which developed a model of population health based on the concept of determinants of health and their interactions (Evans and Stoddart 1990). The CIAR framework offered a synthesis of the evidence on key factors that determine health status including the social environment, the physical environment, genetic endowment, health care, and individual response (including behavior and biology factors, health and function factors, and well-being and prosperity). The fundamental premise of this paradigm was the integration of information from different sources, taking into account all relevant data on the determinants of health as well as the interactions among these risk factors, a component that is essential in understanding the complexities of health (NRC 2011a).

Krewski et al. (2007) developed an integrated approach to risk management and population health that combines key elements of risk science and population health to offer a multidisciplinary approach to the assessment and management of health risk issues, which is critical to fully assess potential human health risks. A key element of this paradigm is the acknowledgment that a complete assessment of a particular risk factor associated with specific adverse health outcome(s) requires consideration of other determinants of those outcome(s) as well as interactions between the risk factor of interest and those determinants. Chiu et al. (2013) proposed a similar approach to advancing human health risk assessment of environmental chemicals that included consideration of a range of health determinants, encompassing chemical stressors as well as nonchemical factors such as genetics, life stage, nutrition, and socioeconomic status that might affect the risks and costs of human disease.

The population health approach to risk assessment emphasizes that determinants of health can interact to affect health status. The powerful interaction between radon and tobacco smoke in the induction of lung cancer—an example of an interaction between the physical and social environments—has implications for risk management policy development (WHO 2009). Approximately 90% of radon-related lung cancer cases occur in smokers and can be eliminated by smoking cessation (NRC 1999). The lung cancer burden from environmental radon could be addressed either by radon mitigation or smoking cessation (or both). This example illustrates the power of the population health approach in expanding the range of risk management options available to deal with important environmental health risks (Krewski et al. 2006, 2009; Turner et al. 2011).

Gene–environment interactions are particularly important in environmental health risk assessment. The National Institute of Environmental Health Sciences (NIEHS) has launched a 5-year program called the Genes and Environment Initiative to investigate the genetic–environmental origin of many common diseases such as cancer, diabetes, and asthma, with the objective of developing new treatments and strategies for prevention (NIEHS 2012). Moore et al. (2010) found an increased risk of renal cancer associated with a genetic allele of glutathione *S*-transferase when individuals were exposed to trichloroethylene (TCE), pinpointing a genetic polymorphism that increases risk depending on the level of environmental exposure to TCE. Population-based studies conducted by Dennis et al. (2011) recently indicated an association between wine consumption and a decreased risk of breast cancer in *BRCA1* (BReast CAncer gene one) mutation carriers and an increased risk in *BRCA2* mutation carriers, documenting an interaction between the genetic and social environments that could have implications for risk management.

## New Risk Assessment Methodologies

The third cornerstone of the NexGen framework is the development and application of new risk assessment methodologies used to characterize population health risks (Table 1). A particularly important contribution in this area is provided by the broad review of chemical risk assessment practices at U.S. EPA that was conducted by the NRC and summarized in the report *Science and Decisions: Advancing Risk Assessment* (NRC 2009). The report recommended a framework for risk-based decision making composed of three phases: *a*) problem formulation and scoping, *b*) planning and conduct of risk assessment, and *c*) risk management. Two themes in the report that are particularly relevant in the NexGen context are *a*) an enhanced role for problem formulation to improve the utility of risk assessment, and *b*) methods for cumulative risk assessment.

**Table 1:** Key risk assessment methodologies for the next generation of risk science: comparison of current and NexGen approaches.

Methodology	Current approach	NexGen approach
Hazard identification, dose–response assessment, and exposure assessment
Hazard identification	Based largely on animal toxicity testing, mainly in rodent species.	Based primarily on *in vitro* testing in human cells, and computational methods in biology and toxicology.
Dose–response assessment	Empirical or biologically based models describe apical end points, and determine an appropriate point of departure (such as the benchmark dose) for establishing a reference dose.	Computational systems biology pathway models describe dose–response relationships for pathway perturbations, reflecting dose-dependent transitions throughout the dose range of interest.
Dose and species extrapolation	Dose and species extrapolation translate animal test results to humans.	Cellular assays provide direct measures of toxicity pathway perturbations in humans. *In vitro* to *in vivo* extrapolation techniques and pathway modeling calibrate *in vitro* and *in vivo* exposures. Sensitive *in vitro* tests are used to evaluate risk directly at environmental exposure levels.
Exposure assessment	Estimates of human exposure based largely on measurements in environmental media (air, food, water, soil).	Expanded use of high throughput biomonitoring data reflecting critical toxicity pathway perturbations.
Characterization of risk and uncertainty
Adversity	Apical outcomes in mammalian systems, or precursors to these outcomes, generally serve as the basis for risk assessment.	*In vitro* assays identify critical toxicity pathway perturbations, which serve as the basis for risk assessment, even in the absence of a direct link with an apical outcome.
Variability	Adjustment factors used in establishing reference doses account for interindividual variability in pharmacokinetics and pharmacodynamics. Variability in exposure is also taken into account.	Variability in biological response is characterized through the use of a diverse range of human cell lines. Dosimetry models link variation in human exposure with corresponding *in vitro* doses.
Life stage and susceptible populations	Life stage, genetics, and socioeconomic and lifestyle factors determine susceptible population groups.	Molecular and genetic epidemiology defines susceptible populations in terms of critical pathway perturbations.
Mixtures and multiple stressors	Common experimental protocols include testing of mixtures and factorial experiments with joint exposures. However, the number of such studies has been limited because of cost and complexity of experimental design.	Cost-effective high throughput technologies permit expanded testing of mixtures and multiple stressors.
Uncertainty analysis	Uncertainty considerations include species differences in susceptibility, low-dose and route-to-route extrapolation, and exposure ascertainment.	Probabilistic risk assessments characterize overall uncertainty, and identify the most important sources of uncertainty that guide value-of-information decisions.

The *Science and Decisions* approach to risk assessment begins with problem formulation, through a preliminary consideration of the risk of interest and the identification of a series of risk management options. The establishment of the risk context improves the utility of the risk assessment process by clearly articulating the overall goals and objectives of the assessment. The NRC (2007) identified five risk decision contexts involving environmental agents: *a)* evaluation of new environmental agents, *b*) evaluation of existing environmental agents, *c*) evaluation of a site, *d*) evaluation of potential environmental contributors to a specific disease, and *e*) evaluation of the relative risks associated with environmental agents.

This perspective on risk-based decision making begins with analyzing current, near-term, and longer-term needs to determine whether the assessment might be done differently in the presence of new data and new methods. Improvements in decision making can come in the form of optimized selection of risk management options, timeliness, resource requirements, transparency, acceptability, and openness to new information or any other desirable attributes of good decision-making processes (NRC 2009).

*Science and Decisions* also provided a series of findings and recommendations that relate to the goal of cumulative risk assessment (NRC 2009). The U.S. EPA is increasingly asked to address broader public-health and environmental-health questions involving multiple exposures, complex mixtures, and vulnerability of exposed populations—issues that stakeholder groups often consider to be inadequately captured by current risk assessments. Because of the complexity of considering so many factors simultaneously, there is a need for simplified risk assessment tools (including databases, software packages, and other modeling resources) to support screening-level risk assessments and possibly to allow communities and stakeholders to conduct assessments (NRC 2009).

## The NexGen Framework: Integration of Three Perspectives

**Figure 1:**
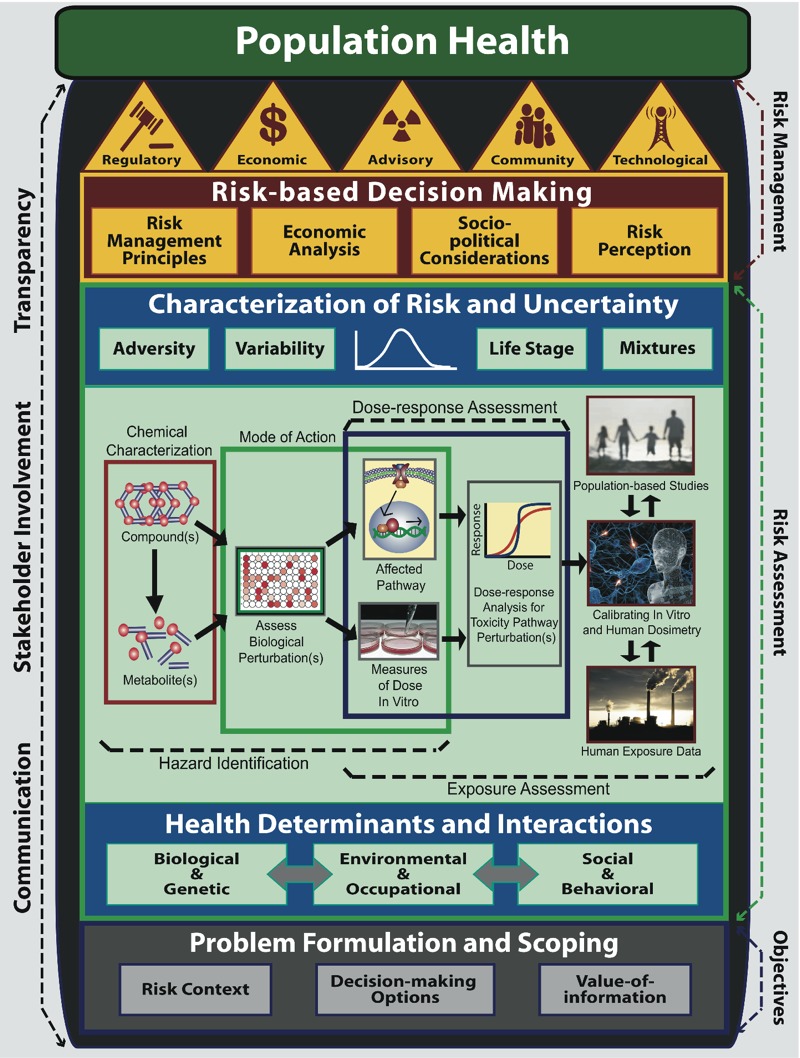
The NexGen framework for risk science. Phase I: objectives—problem formulation and ­scoping takes into consideration the risk context, decision-making options, and value of information. Phase II: risk assessment: health determinants and interactions—incorporates a population health approach that takes into account multi­ple health determinants that interact with the risk factor(s) of interest. Hazard identification, dose–response assessment, and exposure assessment make use of new scientific tools and technologies, based on high throughput screening assays and computational methods in biology and toxicology for hazard identification and dose–response assessment; *in vitro* to *in vivo* extrapolation methods for calibration of *in vitro* and human dosimetry; molecular and genetic epidemiology to identify toxic­ity pathway perturbations in population-based studies; and high-performance mass spectrometry to generate human exposure data, to assess risk. Characterization of risk and uncertainty applies new risk assessment methodologies to develop human exposure guidelines. Phase III: risk management—risk-based decision making considers fundamental risk management principles, economic analysis, socio­political consideration and risk perception to select one or more risk management interventions of a regulatory, economic, advisory, community-based, or technological nature for risk management. [The center section on hazard identification, dose–response assessment, and exposure assessment is adapted from Figure 2 of Krewski et al. (2011).]

The NexGen framework (Figure 1) effectively integrates the three preceding perspectives into an integrated framework for risk science comprised of three phases: Phase I (objectives), Phase II (risk assessment), and Phase III (risk management). After establishing the risk science objectives in Phase I, a scientific assessment of risk using the best available scientific tools and technologies is undertaken in Phase II. Phase III involves the use of scientific evidence in a risk management decision-making context, taking into account extra-scientific considerations such as economic analyses, sociopolitical considerations, and public perception of risk. Risk management decisions, which may involve multiple risk management interventions to reduce risk, are then taken based on fundamental principles of risk management decision making.

### Phase I

*Objectives.* Problem formulation and scoping establishes the overarching goals of the risk assessment and management process. Problem formulation allows for the delineation of admissible risk management options and examination of the value of specific activities in the risk assessment phase, which might help discriminate among these options. The point of problem formulation is to focus risk assessments so that the scientific information that is gathered is cost-effective, is of maximal utility, and encompasses stakeholder concerns. This phase includes consideration of relevant health determinants and their interactions, data gaps that need to be filled, political and industrial costs and impacts, and possible risk management strategies. The objectives phase involves engaging risk managers, risk assessors, and stakeholders early in the risk assessment process to determine the overarching goals, the decision-making context, the timeline, and the depth needed to ensure that the right questions are being asked, as well as the development of a conceptual model and analysis plan for the risk assessment per se. Further discussion of the risk context, decision-making options, and value of information involved is given in Supplemental Material, Phase I: Objectives, pp. 2–3.

The NexGen framework embodies a tiered approach to risk assessment (Cote et al. 2012). Tier 1—screening and prioritization—permits rapid evaluation of literally thousands of environmental agents using high throughput screening (HTS) assays based on *in vitro* tests and quantitative structure–activity relationships. Tier 2—limited scope assessment—involves a more in-depth analysis of hundreds of agents using tier 1 approaches combined with short-term *in vivo* assays in alternative species, medium throughput *in vitro* assays, and extrapolation modeling. Tier 3—in-depth assessment—involves a comprehensive risk assessment of dozens of the highest priority agents using a wide range of toxicity testing approaches, including traditional mammalian toxicology if needed. Because tiers 1–3 involve increasingly complete data on which to base an assessment of risk, residual uncertainties in tier 3 will be notably less than in tiers 1 and 2. Nonetheless, the data available in each tier should be sufficient to support the type of risk decision required for that tier.

### Phase II

*Risk assessment*. The four-stage risk assessment process introduced in the “Red Book,” *Risk Assessment in the Federal Government: Managing the Process* (NRC 1983)—hazard identification, dose–response assessment, exposure assessment, and risk characterization—remains the current benchmark for risk assessment practice. As shown in Figure 1, the risk assessment phase of the NexGen framework expands the classical Red Book framework (NRC 1983) to incorporate a population health approach, new directions in toxicity testing, and new risk assessment methodologies.

Health determinants and interactions encourage consideration of all of the determinants of a health outcome, rather than examining only a single risk factor, as is usually done in traditional risk assessment (Chiu et al. 2013; Krewski et al. 2007). Determinants of health are divided into three categories: biological and genetic, environmental and occupational, and social and behavioral (Figure 1). Biological determinants include factors such as age, sex, and immunocompetence; genetic determinants include genetic polymorphisms resulting in variation in individual susceptibility to environmental exposures, or high-penetrance genes leading to genetic disease in the absence of environmental exposures. Environmental determinants include contaminants present in air, food, water, or soil, as well as the built environment; certain occupations may also result in elevated exposures to chemicals, dusts, and fumes. Social and behavioral health determinants include socioeconomic factors such as education or income, or lifestyle factors such as personal health practices or risk avoidance behavior.

Hazard identification, dose–response assessment and exposure assessment sections illustrate (Figure 1) how the NexGen risk assessment process will be conducted using the tools and technologies within the context of the four-stage risk assessment process introduced in the Red Book.

Hazard identification, the first stage of the risk assessment process, investigates the potential hazard a substance can illicit in humans (NRC 1983). Hazard identification is based on two main components: chemical characterization and mode of action (NRC 2007). As research links physical and chemical properties initially to *in vivo* end points and subsequently to *in vitro* perturbation pathways, structure–activity relationships will become the first tier of screening analysis for predicting the toxicity of a chemical. Subsequent HTS assays, both cellular and cell free, would validate *in silico* predictions and allow for a fuller evaluation of the toxicity of an agent (Rusyn et al. 2012).

Dose–response assessment is the process of characterizing the relationship between the dose of or exposure to an environmental agent and an indicator(s) of health detriment, such as a critical toxicity pathway perturbation or an adverse health outcome observed in exposed human populations. The use of *in vitro* toxicity data for risk assessment depends strongly on the relevance of the *in vitro* data to the *in vivo* context. *In vitro* to *in vivo* extrapolations are necessary to understand the relevance of the compound concentration in both test systems. *In vitro* techniques should represent dose–response relationships for normal physiological functions, adaptive responses, and toxicity pathways, as well as consider variation in epigenetic expression within human populations (Blaauboer 2010; Rotroff et al. 2010; Yoon et al. 2012).

Exposure assessment is the process of measuring or estimating the intensity, frequency, and duration of human exposure to an existing agent, or of estimating hypothetical exposure that might arise from the release of new chemicals into the environment. Although outside the scope of this article, advances in exposure science have recently been discussed in detail by the NRC (2012). The concept of “exposomics,” which integrates a top-down and bottom-up approach to identification of relevant exposure biomarkers, will be an important component of future exposure science (Rappaport 2011).

As the next generation of risk science becomes a reality, new approaches to hazard identification, dose–response assessment, *in vitro* to *in vivo* extrapolation and exposure assessment will be required, including the following (Table 1).

*Hazard identification*. Although *in vitro* test data and epidemiologic studies provide useful information on the toxicity of environmental agents, hazard identification continues to rely heavily the results of animal toxicity tests (NRC 2006). NexGen places greater emphasis on the use of *in vitro* assays in human cells and quantitative structure–activity analysis, as well as the use of computational methods in systems biology (NRC 2007).

*Dose–response assessment*. Quantitative high throughput screening (q-HTS) provides dose–response information over a broad range of test concentrations. The availability of sensitive assays capable of detecting pathway perturbations at very low doses—at or below environmental levels experienced by human populations—will permit characterization of toxicity pathway responses over wide range of doses. Statistical methods can then be used to evaluate benchmark concentrations for adaptive and adverse responses and to assess point-of-departure concentrations (Burgoon and Zacharewski 2008; Parham et al. 2009; Sand et al. 2011; Wignall et al. 2014). As noted above, *in vitro* to *in vivo* extrapolation methods will be required to translate *in vitro* test results to *in vivo* situations using an appropriate internal tissue dose metric (Rotroff et al. 2010; Yoon et al. 2012).

*Dose and species extrapolation*. Low-dose and interspecies extrapolation are two of the long-standing challenges encountered in risk assessment. Various models have supported such extrapolations, including linear and threshold models for low-dose extrapolation and body weight or surface area adjustments for interspecies extrapolation. New extrapolation challenges arise for NexGen assessments based on *in vitro* assay data, including the following: *in vitro* to *in vivo* extrapolation of dosimetry (Blaauboer 2010; Rotroff et al. 2010; Yoon et al. 2012), dose extrapolation of molecular and cellular pathway responses, and extrapolation from the short-term *in vitro* to longer-term *in vivo* exposure periods. *In vitro* to *in vivo* extrapolation and physiologically based pharmacokinetic (PBPK) models are amenable to sensitivity, variability, and uncertainty analysis using conventional tools (Wetmore et al. 2012, 2013). Computational systems biology pathway models of the circuitry and dynamics of pathways will support the application of tools for assessing variability and uncertainty tools from the PBPK literature because the pathway components reflect more targeted molecular constituents and their interactions (Zhang et al. 2010).

*Exposure assessment*. At present, human exposure assessment is based largely on measured levels of environmental agents in the human environment (U.S. EPA 2010); in some cases, internal dose measures may also be obtained using biomonitoring (Hays and Aylward 2008) or pharmacokinetic modeling (Barton et al. 2007). In the NexGen approach, exposure assessment will focus more on direct measures of critical toxicity pathway perturbations in humans by using advanced biomonitoring techniques (NRC 2012) coupled with innovative new high throughput approaches to obtaining indicators of exposure to large numbers of environmental agents simultaneously (Jones et al. 2012).

Further details of the hazard identification and dose–response assessment methods, dosimetry and exposure assessment methods, and cross-cutting assessment methods, that will form the basis of this component of the next generation framework, are provided in Supplemental Material, Table S1, and Supplemental Material, Phase II: Risk Assessment, pp. 3–6.

Characterization of risk and uncertainty involves estimating the rate of occurrence of a health effect associated with a chemical following hazard identification, dose–response assessment, and exposure assessment (NRC 1983). Robust knowledge of how disease mechanisms and toxicity pathways intertwine and operate would eventually allow *in vitro* testing alone to characterize toxicity, at which time the need for *in vivo* testing will be greatly reduced (Rhomberg 2010). The toxicity of a compound should be redefined to include basic chemical and/or biological interactions at the molecular level in a biological system, and risk assessment methodologies will need to evolve as new methods are accepted and validated. Five prominent examples of such methodologies are listed in Table 1 and are discussed below.

*Adversity*. The U.S. EPA defines an adverse effect as “a biochemical change, functional impairment, or pathologic lesion that affects the performance of the whole organism, or reduces an organism’s ability to respond to an additional environmental challenge” (U.S. EPA 2013). With the shift toward biological pathway perturbations as the basis for risk assessment, decisions on which perturbations of cellular response networks are adverse may be made in the absence of information on apical responses in test animals (Boekelheide and Andersen 2010). Such decisions should consider the potentially varying biological context(s) in which such perturbations are seen, as discussed below.

*Variability*. Variability in human response to exposure of environmental agents depends on interindividual differences in the delivery of the agent to target sites (pharmacokinetic differences) and the intensity of responses to the parent compound or metabolites reaching tissue targets (pharmacodynamic difference) (Zeise et al. 2013). Variation in absorption, distribution, metabolism, and excretion (ADME) across individuals determines the range of tissue doses associated with exposure to the agent. Similarly, variation in molecular properties—affinities, concentrations of protective molecules, concentrations of receptors, sensors, and transducer molecules in specific pathways—affect the relative sensitivities of individuals in the population of interest (Simmons et al. 2009). Although variability in pharmacokinetic parameters has received considerable attention, factors affecting tissue response have not. As the factors governing response patterns in toxicity pathways become better understood, this discrepancy will be eliminated, permitting a more complete characterization of both pharmacokinetic and pharmacodynamic sources of variability.

*Life stage and susceptible populations*. Susceptible populations—effectively a manifestation of interindividual variability—have, and will continue to require, special attention in risk assessment. Defining such populations requires an understanding the factors that determine enhanced susceptibility. Lower concentrations of metabolizing enzymes during early life stages could render infants and children more susceptible to certain exposures. Populations with preexisting disease could be more sensitive to exposures that affect already compromised biological function: Exposure to irritant gases and vapors in asthmatics or patient with cardiac or pulmonary disease, for example, would be of greater concern than in otherwise healthy individuals (NRC 2001).

*Mixtures and multiple stressors*. Risk assessment of complex mixtures is complicated by the wide variation in the composition of common mixtures, such as diesel exhaust, and the prohibitive expense of testing a virtually unlimited diversity of such mixtures (U.S. EPA 2001). The speed and reduced cost of *in vitro* assays in assessing multiple mixtures presents opportunities for more comprehensive testing. Current q-HTS platforms greatly facilitate more targeted testing to examine mixtures of chemicals with similar and dissimilar targets affecting a common test assay. Although not high throughput, advances in PBPK modeling provide another approach to assessing interaction among chemicals in mixtures (Haddad et al. 2010; Yang and Andersen 2005).

*Uncertainty analysis*. One of the key risk assessment methodologies discussed by the NRC (2009) is uncertainty analysis. It is important to distinguish between true uncertainty, which is lack of knowledge about one or more risk factors, and variability, which reflects interindividual variation in well-known and easily measurable factors that affect risk, such as body weight. Sophisticated quantitative methods are available to address uncertainties in both the parameters in currently used risk models and uncertainties in the form of the model itself; well-established probabilistic methods can also be used to propagate component uncertainties into an overall uncertainty distribution of plausible risks (symbolically represented by the uncertainty distribution in Figure 1) and to identify the major and minor sources of uncertainty (NRC 2009).

### Phase III

*Risk management*. Risk management is “the process of identifying, evaluating, selecting, and implementing actions to reduce risk to human health and to ecosystems. The goal of risk management is scientifically sound, cost-effective, integrated actions that reduce or prevent risks while taking into account social, cultural, ethical, political, and legal considerations” (Presidential/Congressional Commission on Risk Assessment Risk Management 1997). Risk managers may select a combination of suitable strategies to balance risks, costs and benefits, taking into account social values and political considerations.

Risk-based decision making takes into account well-established principles of risk management, economic analysis, and sociopolitical considerations. Public perception of risk also warrants consideration in the risk management phase.

Risk management principles. Guidance in risk management decision making can be found in fundamental principles of risk management decision making. Jardine et al. (2003) described 10 overarching principles that have evolved over the last three decades: *a*) beneficence and nonmaleficence (do more good than harm), *b*) natural justice (a fair process of decision making), *c*) equity (ensure an equitable distribution of risk), *d*) utility (seek optimal use of limited risk management resources), *e*) honesty (be clear on what can and cannot be done to reduce risk), *f*) acceptability of risk (do not impose risks that are unacceptable to society), *g*) precaution (be cautious in the face of uncertainty, *h*) autonomy (foster informed risk decision making for all stakeholders), *i*) flexibility (continually adapt to new knowledge and understanding), and *j*) practicality (the complete elimination of risk is not possible).

Although each of these principles has significant merit in its own right, the guidance provided by certain principles can lead the decision maker in different directions. Principle *g*, for example, corresponds to the well-known precautionary principle, which, taken in isolation, makes eminent sense: In the face of scientific uncertainty, preemptive risk management action could lead to the avoidance of a major risk disaster. At the same time, principle *d*—the principle of risk-based decision making—suggests that limited risk management resources should be allocated in a manner that will do the most good, by expending available risk management resources on known risks in proportion to the level of modifiable risk.

Balancing the guidance provided by sensible decision-making principles will depend on the risk context, which is set out in Phase I of the NexGen framework. In certain decision-making contexts, some of these principles may not be pertinent. Principle *a*, for example, implicitly counsels a balancing of risks against benefits: Risk–benefit tradeoffs will be admissible in some risk decisions (such as balancing the risks of a serious adverse drug reaction against the possibly lifesaving benefit of the same drug within a patient–physician decision-making context), but not others [such as the prohibition against the use of a direct food additive known or suspected of increasing cancer risk in humans or animals under the Delaney clause in the Food Additives Amendment of 1958, regardless of organoleptic or food processing benefits (FDA 1958)]. In the end, risk decision makers cannot escape invoking a degree of judgment to arrive at an appropriate risk management decision, taking into account all of the scientific and extra-scientific factors that are relevant to the risk decision.

Economic analysis. An economic analysis of implications of alternative risk management options may also be considered in risk management decision making. In its most general form, economic evaluation is the comparative analysis of alternative courses of action, considering both their costs and their consequences (Hoch and Dewa 2005). In the simplest case, one of the alternatives is represented by the status quo, whereas the other alternative is the new program under consideration. Torrance and Krewski (1987) developed a comprehensive framework for the economic evaluation of toxic substance control programs, which includes common economic evaluation approaches such as cost-effectiveness, cost–benefit, and cost–utility analysis as special cases. The incorporation of economic analyses into the risk decision-making process will again depend on the risk context. If benefits are not admissible, cost–benefit analysis will not be useful; for those risk contexts in which monetary benefits are admissible as part of the decision-making process, the major challenge for health economists is to develop valuation estimates for avoided health effects—a challenge that is even more difficult if future risk assessments are based on biological perturbations rather than apical responses. Techniques such as cost-effectiveness analysis avoid the issue of monetization of health benefits, but provide guidance only on the least-cost strategy for exposure reduction, without weighing benefits against costs.

Sociopolitical considerations. Risk management decisions need to take cognizance of social and cultural values, as well as political considerations that may influence the decision-making process. Risk management actions must be acceptable to society at large and respect cultural differences among societal subgroups. The importance of the psycho-social consequences in certain risk decision contexts, such as prion disease risks, is being increasingly recognized (Lemyre et al. 2009). Political constraints will influence risk decisions. At the national level, for example, budget allocation decisions made by governments will dictate the intensity with which the development of regulations and subsequent monitoring and enforcement actions may be pursued. At the international level, mutual recognition and harmonization agreements may influence the choice of risk management actions.

Risk perception. Although often at odds with expert assessments of risk (Krewski et al. 2012), public perception of risk is an important consideration in risk management. Risk perceptions vary by demographic factors including sex, age, and educational attainment, with higher risk perceptions observed among women, older respondents, and respondents with lower educational attainment (Krewski et al. 2006, 2009).

Differences in risk perception between experts and members of the general public can have important consequences for the implementation of risk management and risk communication strategies designed to improve population health. Understanding how the public forms attitudes and opinions about risk and how they might change over time is critical to the design of successful risk communication messages and to public acceptance of and compliance with risk management interventions.

Risk management interventions. The framework for risk management and population health developed by Krewski et al. (2007) emphasizes the use of multiple interventions, rather than relying on a single risk management strategy. Five types of intervention (regulatory, economic, advisory, community, and technological) collectively represent the REACT approach to risk management. The use of regulatory and nonregulatory actions greatly expands the scope of strategies that can be deployed to manage risk. (The decision-making portfolio available to government agencies may be constrained by virtue of the regulatory statues under which they operate; this portfolio may be expanded at the interagency level, with other agencies able to address risk factors that affect or modify the primary risk factor of concern.) Experience with the REACT approach suggests that these five actions span most of the risk management interventions that could be contemplated, and that, taken together, provide a comprehensive suite of options for the mitigation of risk. After multiple interventions are selected and implemented, their impact on population health risk is evaluated, preferably through measurable indicators of population health improvement. Further details on the REACT approach are provided in Supplemental Material, Phase III: Risk Management, pp. 9–11.

As indicated in Figure 1, openness and transparency, stakeholder involvement, and effective communication are essential throughout Phases I, II, and III of the NexGen risk assessment framework.

## Case Study Prototypes

The NexGen project included a series of case study prototypes to evaluate the extent to which new techniques in risk science listed in Supplemental Material, Table S1, are beginning to find application (Table 2). Tier 1 prototypes involve the screening and ranking of tens of thousands of chemical substances (Cote et al. 2012), the tagging of data-poor chemicals by determining the biological pathway altering dose (Judson et al. 2011; Wetmore et al. 2012, 2013), and the ability to make quick decisions in disaster situations such as the Deepwater Horizon oil spill (Anastas et al. 2010). The combination of cataloged data along with HTS allows for the analysis of short-term effects and addresses the question as to which oil dispersant(s) would be most eco-friendly in this environment (Judson et al. 2010). Additional examples of tier 1 profiling include the use of HTS assays for screening endocrine disruptors (Reif et al. 2010) and the use of *in silico* methods to screen and prioritize large numbers of chemicals (Rusyn et al. 2012; Wang NC et al. 2011, 2012).

**Table 2:** New scientific tools and techniques applied (+) or not applied (–) in NexGen case study prototypes.

Scientific tools and techniques	Tier 1		Tier 2		Tier 3	
	Hydrocarbon mixtures and cancer	Oil spill dispersants and endocrine disruption	Chemical exposure and cancer, reproductive, and developmental hazards	Multiple stressors and diabetes	Ozone and lung injury	Benzene and leukemia
Hazard identification and dose–response assessment methods
Quantitative structure–activity models	+	+	+	–	–	–
Toxicity pathway analysis	+	+	+	+	+	+
High throughput *in vitro* assays	–	+	+	+	+	+
High content “omics” assays	–	–	+	–	+	+
Molecular and genetic population-based studies	–	–	–	–	+	+
Biomarkers of effect	–	–	–	+	+	+
Dosimetry and exposure assessment methods
*In vitro* to *in vivo* extrapolation	+	+	+	–	–	–
Pharmacokinetic models and dosimetry	–	+	+	–	+	+
Biomarkers of exposure	–	–	–	+	+	+
Exposomics	–	–	–	–	–	–
Cross-cutting assessment methods
Adverse outcome pathways	+	+	+	+	+	+
Bioinformatics/computational biology	+	+	+	+	+	+
Functional genomics	–	–	+	+	+	+
Systems biology	+	–	+	+	+	+

Tier 2 could involve short-term studies in rodent or nonmammalian species, as well as data mining of human disease databases. Many important pathways are conserved across species and with a pathway-based approach to risk assessment, simple *in vivo* studies such as those done in the fathead minnow, zebrafish, or the nematode *C. elegans* can be used as models for toxicity testing. Perkins et al. (2013) described how different invertebrates could be used as candidates for tier 2 level risk assessment and provide a low-cost alternative to *in vivo* toxicity testing in rodents. Exposure of chemicals from the ToxCast phase I chemical library to developing zebrafish have shown good correlation with toxicity-related end points and cross-species comparisons (Padilla et al. 2012; Sipes et al. 2011). However, Warner et al. (2012) demonstrated markedly different responses of zebrafish and flat head minnow to chemical exposures, reflecting appreciable interspecies variability in sensitivity.

Short-term *in vivo* studies using rodent models could also be used in tier 2 level risk assessment (Table 2). Thomas et al. (2012a) noted that many of the chemicals in contaminated sites are data-poor, and that shorter term studies and an adverse outcome pathway (AOP) approach could solve this problem. AOPs provide a conceptual framework within which to situate specific toxicity pathway perturbations with respect to adversity, with *in vitro* assays for toxicity pathway perturbations serving as the basis for risk assessment. Thomas et al. (2011, 2012b) exposed mice to five different chemicals for 13 weeks and compared classical functional end points with functional genomic microarrays to identify AOPs; results revealed a high correlation between specific AOPs and cancer and noncancer end points.

Another example of tier 2–level assessment explores the question of joint genetic and environmental influences on disease. Research studies have integrated epidemiological, toxicological, and genome-wide association studies and mined the data for risk factors and genetic polymorphisms that influence type 2 diabetes (Patel et al. 2013). This exemplifies the population health approach embedded within the NexGen framework.

Tier 3 assessments—generally required for environmental agents of high concern—have extensive data requirements, including epidemiological, clinical, or traditional animal studies, and the identification of disease-specific toxicological profiles or other mechanistic information (Cote et al. 2012; Wang IM et al. 2012). The tier 3 prototypes include lung injury and ozone (Devlin 2012) and leukemia and benzene (Godderis et al. 2012; McHale et al. 2011, 2012; Thomas et al. 2014). Both prototypes have adopted a systems biology approach and investigate AOPs by comparing “omics” analyses from cells exposed *in vivo* and *in vitro* to detect critical molecular pathway perturbations and intermediate biochemical and pathophysiologic changes leading to adverse apical health effects. The ozone and benzene case study prototypes are based on controlled human experimental data and observational epidemiologic data, respectively; both utilize measured environmental exposure levels. Transcriptomic profiles in lung epithelial cells from human volunteers (exposed to ozone) and peripheral blood mononuclear cells from workers (exposed to benzene) show exposure-dependent alteration in AOPs related to inflammation and lung injury (Devlin 2012) and acute myeloid leukemia (Godderis et al. 2012; McHale et al. 2011, 2012; Thomas et al. 2014). These types of data can provide information on biological mechanisms of action, as well as provide biomarkers of both exposure and biological response. Robust AOP data of this type could be used to screen data-poor chemicals and make inferences about their potential health effects based on mechanistic similarities to data-rich chemicals. Both of these tier 3 risk assessments have used the majority, if not all, of the NexGen tools and technologies discussed in Table S1 in the Supplemental Material. It is anticipated that the NextGen framework for human health risk assessment will evolve over a number of years, with new scientific tools and technologies incorporated into risk assessment practice as they become available. The necessary scientific tools are currently in transition and will shift away from the identification of apical end points in experimental animals toward the identification of critical perturbations of toxicity pathways. The case studies summarized in this article indicate that toxicity testing has already begun moving in this direction.

## Challenges in Implementation

Implementation of NexGen is not without its challenges. Although much of the science on which new approaches to toxicity testing are based is now sufficiently well developed for use in practice, further work is needed to fully characterize toxicity pathways, to develop sensitive and specific high throughput assays to identify critical pathway perturbations, to devise approaches for testing metabolites, and to formalize tools for risk assessment from these studies. Work in this area is currently being done by a number of organizations, notably the National Center for Computational Toxicology at the U.S. EPA (Judson et al. 2011). There are also more expansive approaches such as the “human toxome project” (Hartung and McBride 2011) and more directed case study efforts (Andersen et al. 2011). Until this work provides definitive results, demonstration of adequate margins of exposure relative to levels of biological activity (not all of which is necessarily adverse) could provide assurances of safety (Thomas et al. 2013). During the transition to *TT21C* (NRC 2007), there may also be a need to rely on the results of traditional mammalian toxicity tests, especially when expected human exposures/tissue doses are not much lower than active concentrations from *in vitro* tests.

The shift toward toxicity pathways perturbations in *in vitro* systems as the basis for risk assessment, rather than apical outcomes in experimental animals, presents challenges in predicting potential human health impacts using traditional measures of morbidity and mortality. Health economists may need to develop new indicators of health detriment for use in cost–benefit analysis of alternative risk management strategies. Alternatively, the emphasis of evaluation may become more safety-oriented, focusing on the absence of toxicity pathway perturbations, rather than relying traditional risk assessments based on observed apical responses (Andersen and Krewski 2010; Thomas et al. 2013). This challenge may be overcome in part by population-based studies incorporating molecular markers of pathway perturbations, or possibly by predicting adverse health outcomes on the basis of *in vitro* test results as our understanding of toxicity pathways increases. With the highly sensitive analytical techniques now available to characterize pathway perturbations at very low doses, it should be possible to characterize the shape of the dose–response curve at environmental exposure levels, reducing the need to extrapolate from high to low doses.

In parallel with the recent advances in high throughput approaches to toxicity testing, high throughput mass spectrometry has the potential to greatly enhance our ability to assess human exposure to large numbers of environmental agents simultaneously (Jones et al. 2012). The use of such high throughput approaches not only increases the numbers of environmental agents that can be tested, but also facilitates evaluation of more mixtures of environmental agents. The challenge here will be to develop and validate sensitive and specific measures of pathway perturbations and environmental exposures to encompass biomarkers of both exposure and response.

The adoption of a population health approach, which requires consideration of multiple health determinants affecting the adverse outcome(s) of interest, will lead to an expanded range of risk management interventions. However, current regulatory statutes typically target a specific risk factor, rather than modifying factors, as the basis for risk mitigation. Nonetheless, there may be opportunities to exploit broader risk management strategies targeting multiple health determinants in situations where a cross-agency risk management solution is possible.

## Conclusions

Toxicity testing is undergoing a transformation toward a new paradigm that will require changes in the practice of risk assessment. The NRC (2007) has articulated a long-term vision for toxicity testing that has received widespread support, both within the United States and internationally. Although the toxicity testing methods envisaged by the NRC (2007) as the scientific toolbox for toxicity testing in the 21st century will differ notably from those currently in use, they are still compatible with the well-established risk assessment paradigm laid out in the 1983 Red Book (Krewski et al. 2011; NRC 2007). The implications of this vision for future risk assessment practice have been the subject of constructive debate (Blaauboer 2010; Krewski et al. 2011). Our initial exploration of these implications suggests that changes in risk assessment practice will be required in order to properly evaluate the new types of toxicity data that are emerging within the context of the NexGen framework.

The original 2007 NRC vision for the future of toxicity testing laid out a 10- to 20-year timeline for the shift toward toxicity pathway–based risk assessment to be realized in full. Since that time, progress toward this goal has been made more rapidly than expected, thereby facilitating the adoption of next generation framework for risk science outlined in this article. Recently, the Council of Canadian Academies (2012) has taken stock of scientific advances supporting the NRC vision and identified tools and technologies currently available as well as those that may be reasonably anticipated to come online within the near term (2–10 years).

The NexGen framework as articulated here integrates three complementary perspectives on human health risk assessment: *a*) the NRC report *Toxicity Testing in the 21st Century: A Vision and a Strategy* (NRC 2007); *b*) a population health approach to risk assessment (Chiu et al. 2013; Krewski et al. 2007); and *c*) the adoption of new risk assessment methodologies, such as those in the NRC report *Science and Decisions: Advancing Risk Assessment* (NRC 2009). The NexGen framework will transform human health risk assessment from a process that has focused on a small number of chemicals, relying primarily on apical end points, to one that manages the majority of chemical exposures by characterizing the risk of critical toxicity pathway perturbations. With the recent advances in molecular and genetic epidemiology, population-based studies may assume greater prominence within the NexGen framework by virtue of their ability to identify perturbations of toxicity pathways directly in human populations at environmental exposure levels The incorporation of a population health perspective taking into account multiple determinants of health and the interactions among them will not only enhance our understanding of disease etiology and adverse outcomes, but may also expand the range of risk management options for dealing with critical health risk issues. Sound principles of risk management decision making will permit the identification of multiple risk management interventions designed to reduce population health risks within the context of the NexGen framework for risk science.

## Supplemental Material

(179 KB) PDF
