# Supplementary material for: A Framework for the Next Generation of Risk Science
Source: Environ Health Perspect. 2014 Apr 11;122(8):796–805. doi: 10.1289/ehp.1307260 (PMC4123023; doi:10.1289/ehp.1307260)
Supplement: (179 KB) PDF [file ehp.1307260.s001.pdf]

Environmental Health Perspectives doi:10.1289/ehp.1307260

Supplemental Material

A Framework for the Next Generation of Risk Science

Daniel Krewski, Margit Westphal, Melvin E. Andersen, Gregory M. Paoli, Weihsueh A. Chiu, Mustafa Al-Zoughool, Maxine C. Croteau, Lyle D. Burgoon, and Ila Cote

Table of Contents **Page**

A framework for the next generation of risk science **2**

Phase I: Objectives ***2***

Phase II: Risk assessment ***3***

Hazard identification and dose-response assessment methods 3

Dosimetry and exposure assessment methods 4

Cross-cutting assessment methods 5

Table S1. Applications of key scientific tools and techniques in the next generation of risk science **7**

Phase III: Risk management ***9***

Regulatory interventions 9

Economic interventions 9

Advisory interventions 9

Community interventions 10

Technological interventions 10

References **12**

A framework for the next generation of risk science

Krewski et al. (2014) introduced a new framework for the next generation of risk science comprised of three phases (Phase I, Problem Formulation and Scoping; Phase II, Risk Assessment; and Phase III, Risk Management). Additional details on each of these three phases are given below.

Phase I: Objectives

Problem Formulation and Scoping begins by examining the risk context and identifying a series of risk management options, thereby streamlining the assays required for risk characterization (NRC 2009). The establishment of the risk context improves the utility of the risk assessment process by clearly articulating the overall goals and objectives for risk analysis. The risk context also determines how detailed an analysis is required, whether tens of thousands of chemicals need to be screened or categorized, or whether intensive and complex testing is required for a smaller number of high priority chemicals. The data required will depend on whether the ultimate objective is to establish a human exposure guideline that avoids the occurrence of toxicity pathway perturbations, or to characterize potential human health risks at higher exposure levels. As our understanding of toxicity pathways increases, it may be possible to predict the risk of adverse health outcomes based on *in vitro* data.

In Phase I, consideration needs to be given to decision-making options, considering current, near-term, and longer term risk management objectives. By considering the risk management objectives carefully at the outset, it will be possible to tailor the risk assessment approach to those objectives. This will result in the design of an efficient risk assessment strategy that will optimize the selection of the most appropriate risk management strategies in Phase III.

Value-of-information (VOI) distinguishes data from information by focusing on whether a specific piece of data has an impact on decisions. VOI analyses may be conducted in the presence of preliminary risk assessment information, taking into account uncertainties in the available data. (In the absence a quantitative uncertainty analysis, the NRC (2009) recommends consideration of a less formal VOI analysis.) Information systems can provide support for a portfolio of decisions, and the suite of new scientific and technological tools will yield data and analyses that constitute a new form of information system that informs decision-making (NRC 2009).

Phase II: Risk assessment

The key scientific tools and technologies that will form the core of NexGen risk assessments are summarized in Supplemental Material, Table S1, below.

Hazard identification and dose-response assessment methods

Currently, apical responses in intact animals or in human populations generally form the basis for deciding which responses are hazardous and at what dosage. Confidence in using pathway perturbations as the basis of or as supporting information for risk assessment will be strengthened as our understanding of toxicity pathways increases (NRC 2007). A full understanding of toxicity pathways is likely to require a 'human toxome project' (Hartung & McBride 2011) on the scale of the now complete human genome project.

Some sources of uncertainty—such as uncertainty associated with extrapolation from high to low doses—will be mitigated by the use of sensitive *in vitro* assays that can be used to estimate risk directly at environmental exposure levels. Emerging risk assessment methodologies for application with *in vitro* data, such as the BPAD (biological pathway activating dose) approach

proposed by Dix et al. (2012), explicitly incorporate both uncertainty and variability in the analysis.

Dosimetry and exposure assessment methods

Toxic responses depend on integrated, often complex, interactions of molecular networks that involve hundreds or even thousands of genes, proteins, metabolites, and distinct pathways. The host genome and epigenome also influence the level of response for these molecular networks (Olden et al. 2011). Environmental factors—collectively labelled the 'exposome'—are believed to contribute significantly to human disease risk (Rappaport 2011; Wild 2005).

Two different strategies have been proposed to evaluate the exposome (Rappaport 2011). The first is a 'bottom-up' approach, where chemicals are measured in air, food, water, and other external sources. Estimation of environmental exposures can been done using questionnaire data describing occupational and environmental exposure situations, as well as probabilistic modeling. In order to obtain a complete exposure profile for the agent or agents of interest, all sources of exposure should be considered. *In silico* methods can be used to predict absorption, distribution, metabolism, and excretion in mammalian systems, thereby providing tissue dosimetry in support of risk assessment. These models can be used to integrate *in vitro* metabolic data to predict dose- and species-dependent *in vivo* effects (Rietjens et al. 2010).

The second or 'top-down' approach measures chemical, biomarker, and signature profiles in serum and blood. This type of analysis identifies important bio-signals that may predict increased disease risk. Internal changes in tissue dosimetry result from external exposures (such as air and food), lifestyle (including factors such as diet and smoking) and endogenous sources (inflammation, infection, and other factors) (Rappaport 2011). New technology such as high

resolution mass spectrometry can analyze many thousands of metabolites from just a small sample of plasma (Jones et al. 2012).

The National Health and Nutritional Examination Survey (NHANES) is an ongoing population study that assesses the health and nutritional status of the general population. Biomarkers for different exposures are measured in the blood and urine of these subjects, and several reports have been written analyzing the results (CDC 2012). Biomonitoring equivalents (BEs) were proposed as a structured approach to employing existing pharmacokinetic data to bridge the gap between the traditional toxicology and chemical risk assessment paradigm (Hays and Aylward 2008). The biomonitoring equivalent approach uses existing pharmacokinetic data to estimate concentrations of biomarkers that are associated with a range of exposure levels of risk assessment interest.

Cross-cutting assessment methods

Computational systems biology—which involves the development and application of data-analytical and theoretical methods, mathematical modeling and simulation techniques to the study of biological systems—will play a major role in integrating all of the relevant information for purposes of risk characterization. Systems biology provides a basis for incorporating the complex analyses required to determine whether a biological system could maintain homeostasis or trigger adverse outcome pathways (AOP) that lead to adverse health outcomes (Blaauboer 2010; Krewski et al 2011; Rhomberg 2010; Zhang et al. 2010). As test platforms for q-HTS expand, the analysis of chemical mixtures, susceptibility at different life-stages, and influence of health determinants on different possible exposure circumstances will become possible (Kavlock et al. 2012). Computational toxicology makes use of *in silico* methods involving advanced

computational methods to solve a biologically based mathematically models to predict the toxicity of environmental agents (Zhang et al. 2010).

Table S1. Applications of key scientific tools and techniques in the next generation of risk science.

**Scientific tools and techniques**	**Description and application**
**Hazard identification and dose-response assessment methods**	
Quantitative structure-activity relationships (QSAR)	QSAR predicts toxicological responses and metabolic pathways based on the chemical properties of environmental agents and comparison with other active structures.
Toxicity pathway analysis	Toxicity pathway analysis involves the use of human cells and cell lines to assess biological pathway perturbations based on specific or generic modes of action. A suite of these assays could form the test battery for safety assessment.
High throughput *in vitro* assays	High throughput *in vitro* assays are used to rapidly describe concentration response curves for multiple toxicity pathway endpoints, across a broad range of concentrations for large numbers of compounds.
High content 'omics' assays	Transcriptomic, proteomic, metabolomic, micro-RNA, and epigenetic (DNA-methylation) platforms are used to assess perturbations in cellular and tissue function.
Molecular and genetic population-based studies	Population-based studies incorporating molecular markers of exposure and biological change integrate knowledge of the human genome into epidemiological studies to better understand the roles of genetic susceptibility and gene-environment interactions in disease causation.
Biomarkers of effect	Biochemical or molecular markers that correlate with expected biological responses in cells, individuals or populations and may be linked to toxicity pathway perturbations, thereby providing direct evidence of critical perturbations in human populations.
**Dosimetry and exposure assessment methods**	
In vitro to *in vivo* extrapolation (IVIVE)	Toxicokinetic factors, such as protein binding, liver/kidney clearance and oral uptake, can be used to translate *in vitro* doses to *in vivo* exposures thereby permitting the use of *in vitro* data for human safety assessment.
Pharmacokinetic models and dosimetry	Physiologically based pharmacokinetic models are used to understand the absorption, distribution, metabolism, and elimination of environmental agents. Dosimetric methods are used to extrapolate between different exposure routes and dosing regimens, and characterize inter-individual variability in exposure and dose.
Biomarkers of exposure	Biochemical or molecular markers of exposure in blood, urine, breath or other matrices can be compared with biomarkers of effect to evaluate margins of exposure in populations of interest. High resolution mass spectrometry methods are now able to measure thousands of metabolites in blood and other matrices, supporting the assessment of exposure to large number of environmental agents simultaneously.
Exposomics	Exposomics considers the totality of environmental exposure from conception throughout life to provide a better understanding of human diseases through knowledge of the internal chemical environment of individuals.

Scientific tools and techniques	Description and application
**Cross-cutting assessment methods**	
Adverse outcome pathways (AOPs)	AOPs, which describe the sequence of biological events from a molecular initiating event (MIE) through to the development of an adverse health outcome at the individual or population level, provide a conceptual framework in which specific toxicity pathway perturbations can be situated.
Bioinformatics/computational biology	Methods in bioinformatics and computational biology can be used to interpret complex multivariable data from quantitative high throughput screening (q-HTS), high content imaging (HCI), and genomic assays to identify modes of action and predict effects of sustained toxicity pathway perturbations on organs and tissues using mechanistic models at the cellular and molecular level.
Functional genomics	Functional genomics is used to integrate diverse 'omic's information, including proteomics, metabolomics, transcriptomics, epigenetics, and micro RNAs to understand the consequences of pathway perturbation for the cell, organ, and organism.
Systems biology	Systems biology provides the tools needed to organize information from multiple cellular response pathways to understand integrated cellular and tissue responses, and characterize dose-response behaviors of the system based on perturbations of network circuitry by environmental agents.

Phase III: Risk management

Risk managers may choose one or more interventions to address the risk issue of concern. The five major types of intervention that are typically considered in practice are described below.

Regulatory interventions

Government agencies responsible for risk management rely heavily on regulatory action to eliminate or reduce risk. While regulatory action is an essential component of risk management (depending on the regulatory statues governing a particular risk issue, regulation may be the only option for managing risk), non-regulatory solutions to critical risk issues can be equally or more effective than regulatory solutions. The costs associated with regulatory solutions can be high and alternative solutions are often more cost-effective (WHO 2000).

Economic interventions

Economic interventions generally involve economic incentives or disincentives that lead to the reduction of risk. For example, economic incentive programs such as financial assistance or subsidies can expedite development and acceptance of superior, lower risk technologies. Economic disincentive programs include liability insurance, which provides compensation to injured parties. The Price-Anderson Act, for example, requires nuclear power facilities to contribute pay insurance premiums to cover damages in the extent of a nuclear mishap (U.S. NRC 2012).

Advisory interventions

The advisory approach to risk management essentially relies on the provision of timely information to individuals and groups at risk so that they can make informed decisions regarding personal risk behaviors. This information can also be targeted at risk producers to encourage risk

reduction or toward risk consumers to promote risk avoidance. Providing people with information on how to avoid coming into contact with the HIV virus represents a highly cost-effective advisory approach to risk management (Cohen et al 2004).

Community interventions

Grass roots community action can contribute to health risk in different ways. Community action groups can assist in setting priorities, proposing risk management options, and planning and implementing health-related initiatives. For example, the goal of Toxic-Free (http://www.toxicfreecanada.ca/), a volunteer, multi-sector, community-based group, is to reduce the use of toxic household products and to encourage the public to use environmentally safe products for cleaning. These groups have broad influence and are an effective means of changing behaviors. Community action can also contribute to population health through the initiation of health promotion programs targeting healthy lifestyle changes.

Technological interventions

Technological interventions exploit different technologies to prevent or reduce risk. A technological approach could be part of a regulatory initiative and could be as simple as installing newer technology or repairing old technology. The development of activated-carbon filters to absorb organic compounds, for example, has increased air and water quality and reduced health risk in the absence of regulatory pressures (Ao and Lee 2005; WHO 2014).

A full treatment of risk decision analysis and principles of risk management decision making is outside the scope of this paper, the primary purpose of which is to chart the future of risk science as described in Phase II (Risk Assessment). The inclusion of Phases I (Problem Definition) and III (Risk Management) is intended to situate advances in risk science within the broader context

of risk management decision making, and to illustrate the linkages between Phases I, II, and III of the NexGen risk assessment paradigm.

References

Ao CH, Lee SC. 2005. Indoor air purification by photocatalyst TiO2 immobilized on an activated carbon filter installed in an air cleaner. Chem Eng Sci 60:103-109.

Blaauboer BJ. 2010. Biokinetic modeling and *in vitro-in vivo* extrapolations. J Toxicol Environ Health B Crit Rev 13:242-252.

Cohen DA, Wu S-Y, Farley TA. 2004. Comparing the cost-effectiveness of HIV prevention interventions. J Acquir Immune Defic Syndr. 37:1404-1414.

CDC (Centers for Disease Control and Prevention). 2012. The National Health and Nutrition Examination Survey (NHANES). Available: http://www.cdc.gov/nchs/nhanes.htm [accessed 31 January 2014].

Dix DJ, Houck KA, Judson RS, Kleinstreuer NC, Knudsen TB, Martin MT, et al. 2012. Incorporating biological, chemical and toxicological knowledge into predictive models of toxicity. Toxicol Sci 130:440-441.

Hartung T, McBride M. 2011. Food for thought ... on mapping the human toxome. ALTEX 28:83-93.

Hays SM, Aylward LL. 2008. Using biomonitoring equivalents to interpret human biomonitoring data in a public health risk context. J Appl Toxicol 29:275-288.

Jones DP, Park Y, Ziegler TR. 2012. Nutritional metabolomics: progress in addressing complexity in diet and health. Annu Rev Nutr 32:183-202.

Kavlock R, Chandler K, Houck K, Hunter S, Judson R, Kleinstreuer N, et al. 2012. Update on EPA's ToxCast program: providing high throughput decision support tools for chemical risk management. Chem Res Toxicol 25:1287-302.

Krewski D, Westphal M, Al-Zoughool M, Croteau MC, Andersen ME. 2011. New directions in toxicity testing. Annu Rev Public Health 32:161-178.

Krewski D, Westphal M, Andersen ME, Paoli G, Chiu W, Al-Zoughool M, et al. 2014. A framework for the next generation of risk science. Environ Health Perspect. To appear.

NRC (National Research Council). 2007. Toxicity testing in the 21st century: a vision and a strategy. Washington, DC: National Academy Press.

NRC (National Research Council). 2009. Science and decisions: advancing risk assessment. Washington, DC: National Academy Press.

Olden K, Isaac L, Roberts L. 2011. Neighborhood-specific epigenome analysis: the pathway forward to understanding gene-environment interactions. N C Med J 72:125-127.

Rappaport SM. 2011. Implications of the exposome for exposure science. J Expo Sci Environ Epidemiol 21:5-9.

Rhomberg LR. 2010. Toxicity testing in the 21st century: how will it affect risk assessment? J Toxicol Environ Health B Crit Rev 13:361-375.

Rietjens IM, Punt A, Schilter B, Scholz G, Delatour T, van Bladeren PJ. 2010. *In silico* methods for physiologically based biokinetic models describing bioactivation and detoxification of coumarin and estragole: Implications for risk assessment. Mol Nutr Food Res 54:195-207.

U.S. NRC (United States Nuclear Regulatory Commission). 2012. Fact sheet on nuclear insurance and disaster relief funds. Available: http://www.nrc.gov/reading-rm/doc-collections/fact-sheets/funds-fs.html [accessed 28 January 2014].

WHO (World Health Organization). 2014. Water Sanitation Health: Managing water in the home. Available: http://www.who.int/water_sanitation_health/dwq/wsh0207/en/index6.html [accessed 4 February 2014].

WHO (World Health Organization). 2000. Considerations in evaluating the cost-effectiveness of environmental health interventions. Available: https://extranet.who.int/iris/restricted/handle/10665/66744 [accessed 25 March 2014].

Wild CP. 2005. Complementing the genome with an "exposome": the outstanding challenge of environmental exposure measurement in molecular epidemiology. Cancer Epidemiol Biomarkers Prev 14: 1847-1850.

Zhang Q, Bhattacharya S, Andersen ME, Conolly RB. 2010. Computational systems biology and dose-response modeling in relation to new directions in toxicity testing. J Toxicol Environ Health B Crit Rev 13:253-276.
